# Vet Connect: A Quality Improvement Program to Provide Telehealth Subspecialty Care for Veterans Residing in VA-Contracted Community Nursing Homes

**DOI:** 10.3390/geriatrics3030057

**Published:** 2018-09-05

**Authors:** Anne Hale, Leah M. Haverhals, Chelsea Manheim, Cari Levy

**Affiliations:** 1 VA Eastern Colorado Healthcare System Center of Innovation for Veteran-Centric and Value-Driven Care, 13611 East Colfax Ave., Aurora, CO 80045, USA; leah.haverhals@va.gov (L.M.H.); chelsea.manheim@va.gov (C.M.); cari.levy@va.gov (C.L.); 2 School of Medicine, The University of Colorado Denver, Aurora, CO 80045, USA

**Keywords:** healthcare models, geriatric care, telehealth programs, cost savings

## Abstract

Veterans residing in Veterans Health Administration (VA) contracted Community Nursing Homes (CNHs) receive primary care from the CNH they reside in, but often travel to Veterans Affairs Medical Centers (VAMCs) for specialty care services. The Vet Connect project is a quality improvement project aiming to implement video technology to support access to specialty care. **Methods**: Eight Denver VAMC specialty care providers and three project nurses underwent telehealth training and obtained appropriate equipment. To identify in-person visits eligible for substitution of video visits, project nurses review charts of CNH Veterans, consult directly with Veterans, and obtain recommendations from staff. Project nurses serve as tele-presenters within the CNHs, while VA specialists provide care from the VAMC. After each visit, team nurses coordinate care with and deliver specialty care recommendations to CNH staff. **Results**: We assessed clinical, business, and technical domains of the Vet Connect project, and utilized process mapping to identify barriers and facilitators to implementation. Clinically, starting on 26 June 2017 through 1 June 2018, *N* = 203 video visits have been conducted with 11 different CNHs in three subspecialties: geriatrics, palliative care, and mental health. These visits generated 49 referrals for 37 Veterans. Fiscally, cost analyses indicate that per visit, the health care system saves an estimated $310. Technologically, the success rate was 83%. Process mapping helped identify facilitators and barriers to implementation of the telehealth program, including cultivating buy-in from key stakeholders (i.e., medical and mental health providers, telehealth staff, and CNH staff), communication allowing for ongoing program adaptation, and building relationships. **Conclusion**: Subspecialty care delivery to nursing homes using video visit technology in the Vet Connect program is feasible using centralized organization to coordinate complex clinical, business and technical processes. Vet Connect has proved sustainable and has potential to expand within and outside of the VA.

## 1. Introduction

Telehealth services are beneficial for those who reside in rural communities and those who find travel of any distance to a medical center difficult [1]. Telehealth programs increase access and efficiency of care while decreasing cost and travel time associated with obtaining healthcare [2,3]. One such Veterans Health Administration (VA) program demonstrated feasibility in 2013 by providing over 600,000 Veterans Clinic Based Video Telehealth (CVT), real-time video consultation, and appointments in over 44 clinical specialties [4]. Another VA program serving chronically-ill, primarily aging Veterans at home, called Care Coordination and Home Telehealth program (CCHT), demonstrated success and program value. This included a 25% reduction in inpatient bed days of care, a 19% reduction in hospital admissions, reductions in cost of care (the authors noted annual per patient CCHT costs of $1600), and a mean satisfaction score of 86% for Veterans enrolled in the program [5]. 

These efforts are consistent with the VA Strategic Plan for 2013 to 2018 which states an overall objective to provide Veterans personalized, proactive, patient-driven health care [6]. To accomplish this objective, several sub-objectives were proposed, including innovation and improvement, which encompass advancing emerging technologies, as well as strengthening collaboration with the community. Based on these objectives and an increased interest in and demand for telehealth services, a goal emerged: To offer VA specialty telehealth care to any location, including the Veteran’s home or non-VA facilities. The intent is to serve Veterans who are not able to travel easily to a VA Medical Center (VAMC) or VA Community Based Outpatient Clinic (CBOC), due to health issues or rurality. More recently, additional efforts have been made to increase access to telehealth through the VA Mission Bill [7], whereby Veterans who desire a specialty telehealth appointment may access providers within or across state lines. 

We implemented the current quality improvement project to provide VA specialty medical care and mental health support via telehealth to a subgroup of Eastern Colorado Health Care System (ECHCS) Veterans who reside in contracted Community Nursing Homes (CNHs). The few studies published providing elderly or nursing home care via telehealth suggest positive health outcomes, cost benefits, and staff satisfaction for patient glycemic management [8]; rehabilitation after a stroke, fall, fracture, or prolonged hospital admission [9]; continence assessment and management [10]; after hours primary care [11]; and physical therapy [12]. Evidence is also beginning to demonstrate that tele-psychiatry is a viable substitute for face-to-face visits to treat depression in elderly patients [13]. Specifically, within VA, a telehealth program implementing video visits between five VAMCs and their associated community-based facilities found individually tailored therapeutic strategies through telehealth proved to be an effective strategy in the treatment of post-traumatic stress disorder (PTSD) [14]. 

While literature shows benefits of telehealth to the geriatric population [15], and recent research with clinicians and nursing home administrators has demonstrated desire to use telehealth to meet unmet needs of nursing home residents [16,17], to date expansion of such care remains sparse, especially for nursing home residents. The Vet Connect project aimed to fill this void. Vet Connect is a part of a larger effort to increase access to specialty care services for Veterans living in long-term care (LTC) facilities in Oklahoma and Colorado. Upon closure of the Denver VAMC’s nursing home (called the Community Living Center or CLC) in April 2017, we designed this project as a telehealth service to provide longitudinal follow-up for Veterans who had resided in the CLC and transitioned to CNHs. In June 2017, implementation of Vet Connect began, serving the needs of CNH Veterans in the Denver area. As the VA has over 30,000 Veterans receiving contracted nursing home care, and this need is expected to expand to over 45,000 Veterans by 2030 (a 50.4% increase) programs like Vet Connect have great reach and potential to expand [18].

The aims of this paper are to: (a) Describe the implementation process of this quality improvement project delivering telehealth to geriatric Veterans; (b) explain facilitators and barriers encountered during implementation; (c) report utilization and cost measures; and (d) discuss the refined process and plans for sustainability of the Vet Connect program. 

## 2. Materials and Methods 

**Figure A1:**
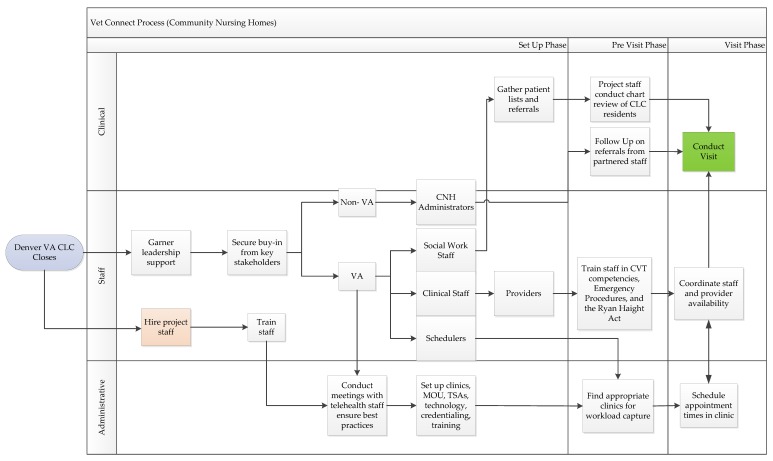
Process of Vet Connect development.

**Figure A2:**
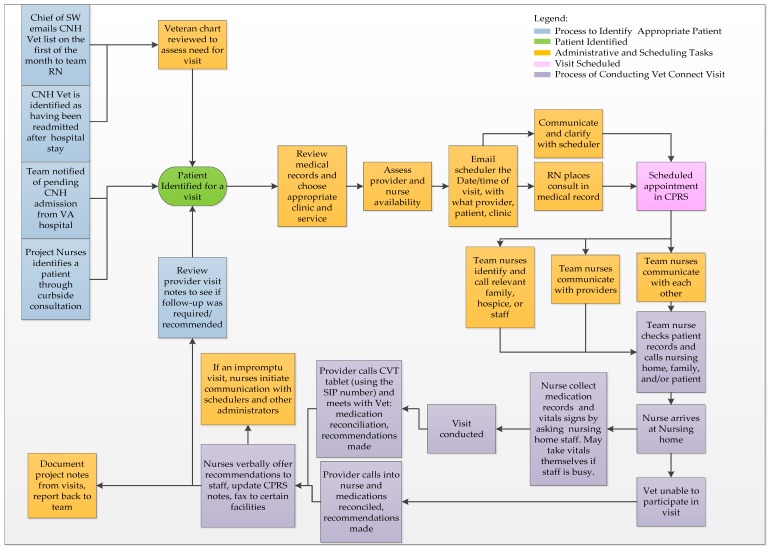
Process of video visit veteran identification, scheduling, commencement, and post-visit procedure.

The following materials and methods were utilized in implementation of Vet Connect; staffing, stakeholder engagement, administrative efforts, technology, and processes for visit preparation and commencement and post-visit follow-up. To illustrate materials and methods, our team created process maps A1 and A2 to clearly identify processes, barriers, and facilitators of program implementation; Figure A1 illustrates Section 2.1, Section 2.2 and Section 2.3, while Figure A2 illustrates Section 2.5 and Section 2.6. We used micro-costing techniques to estimate the financial resources saved from delivering a video visit as compared to delivering a visit face-to-face and is discussed in the results section [19]. Prior to project commencement, the project team procured a Quality Improvement designation letter from operational partners. 

### 2.1. Staffing 

Vet Connect initially provided specialty care services to CNH Veterans within geriatrics and palliative care. We then identified mental health as a needed service, and providers in palliative care mental health and geriatric mental health were added to the program. Currently, Vet Connect partners with two geriatricians (one who specializes in dementia); two psychologists (one who specializes in palliative care); three palliative care specialists; and one psychiatrist. Three part-time nurses act as tele-presenters during CNH visits. They coordinate care prior to and following visits by reviewing clinical issues with the CNH staff, obtaining a list of medications, and facilitating care coordination after visits. Importantly, all staff involved (except one psychologist and the project psychiatrist) previously worked at the Denver CLC prior to its closing, thus relationships between many staff and many Veterans within the program already existed. 

### 2.2. Stakeholder Engagement

To secure buy-in from key stakeholders, we visited CNHs in person to ascertain needs and generate communication between CNH and VA staff. We also ensured the telehealth services offered filled gaps in care without duplicating existing services. In addition, we conducted meetings with VA staff, providers, social workers, and nurses to coordinate efforts and generate partnerships at multiple time points during the first year of Vet Connect. 

### 2.3. Administrative

Telehealth leadership at the Denver VAMC assisted in the creation of appropriate clinics and consult notes and developing Memorandums of Understanding and Telehealth Service Agreements (both documents outline responsibilities and processes associated with the telehealth program) between the VAMC and CNHs. Training and credentialing providers ensured appropriate implementation of services. In accordance with contracts between the VAMC and CNHs, primary care is provided by the CNH, while other specialty care is provided by the VAMC.

### 2.4. Technology

Denver VAMC telehealth program specialists educated the project team on use of CVT tablets to conduct visits between the CNH and the VAMC. To initiate a visit, a team provider dials the specified CVT tablet number into their computer enabled with Jabber software [20]. Meanwhile at the CNH, the Veteran, or supporting staff simply accept the incoming call from their provider by tapping on a green icon on the CVT screen. 

### 2.5. Visit Preparation and Commencement

There are several steps involved to prepare for a visit. Nurses identify suitable Veterans for a video visit (either through direct consultation, identification from VA social workers, or through VA or non-VA staff referral). Suitable Veterans are defined as those Veterans who are in VA CNHs, who have or need a face to face visit that could be substituted for a video visit, and whose provider agrees to a substitution. Furthermore, as the project progressed, Veterans with cognitive impairments such as dementia were deemed inappropriate for video visits as communication was difficult. The process and preference for video over phone appointments, especially in building rapport and reading facial expressions, has been preferred in past research [21]. 

After coordinating times between team nurses, provider, Veteran, and in some cases, the Veteran’s family, the visit time and date is scheduled. Team nurses will arrive at the CNH an hour prior to the scheduled visit to obtain patient vitals and medical information to best assist as the eyes, ears, and hands of the remote specialist. At the appropriate time, the nurse meets with the Veteran in the CNH, in a private and quiet setting, and the provider calls into the CVT tablet using Jabber software [20]. 

### 2.6. Post-Visit Process

First, a team nurses update the Veteran’s VA electronic medical records and input any referrals or follow-up appointment consultations that were generated during the visit. Second, the team nurses communicate to the CNH staff the recommendations from VA providers either in person or through fax. Lastly, visits and field notes are tracked in a secure database. 

### 2.7. Cost Analysis

We used micro-costing techniques to estimate the financial resources saved from delivering a video visit as compared to delivering a visit face-to-face [19]. We engaged VA and CNH stakeholders to identify sub-activities and cost domains for each visit format. After cost domains were agreed upon by VA and CNH stakeholders, we held key informant interviews with individuals at the VA and CNH to identify units and unit costs for each cost domain. Cost domains were summed for each visit format to calculate the average cost of delivering a visit face-to-face and the average cost of delivering a video visit. Cost estimates were presented from two perspectives: VA and CNH. Cost avoidances associated with delivering a video visit were calculated from both perspectives and from the health system in total by differencing the cost between the face-to-face visit and video visit. 

## 3. Results

We defined outcomes in terms of the following domains: clinical utilization, business, and technical. We also created process maps to best assess pre- and post-visit processes, as well as provide guidance for future implementation efforts. Vet Connect model outcomes during the 11 months following implementation are described by domain below in Section 3.1, Section 3.2, Section 3.3 and Section 3.4. 

### 3.1. Clinical Utilization

**Figure 1:**
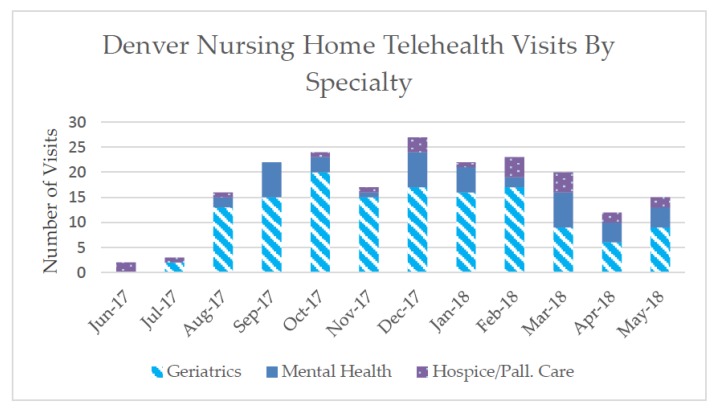
Video Visits by Sub-Specialty June 2017–May 2018.

We conducted 203 visits in 11 different CNHs, between 26 June 2017 and 1 June 2018 (see Figure 1). Specialties included hospice and palliative care, mental health, and geriatrics, with geriatrics the most frequently provided type of subspecialty visit. Follow-up visits accounted for 22.2% of these visits (*N* = 45). Referrals for ancillary services or other medical or mental health treatments totaled 49 referrals for 37 Veterans. Video visit times ranged from 10 to 60 min. 

### 3.2. Cost Analysis

Cost domains included: Staffing time to coordinate travel and schedule appointments; transportation costs; video visit technology including CVT tablets and provider video cameras; and office space. Based on these domains, the cost of delivering a visit face-to-face was approximately $187.34 for the VA and $171.01 for the CNH. The cost of delivering a video visit was approximately $30.67 for the VA and $5.70 for the CNH. Therefore, for every video visit substituted for a face-to-face visit, CNHs’ cost savings are estimated at $160, and the VA’s cost savings are estimated at $150. Thus, in total, the health care system saves an estimated $310 per video visit substituted for a face-to-face visit.

### 3.3. Technical

Vet Connect visits to date have an 83.3% technical success rate (169 successful visits compared to 203 total visits). This is defined as a visit with zero reported technical difficulties: No dropped calls or reports of poor connectivity such as screen freezes and audio lag. Primary reasons for technology failure included poor connectivity using the tablet’s 4G data plan and Veteran difficulty hearing the provider. External headphones were added to enhanced volume for Veterans with hearing impairment. During 12 of the 34 visits with technical difficulties, nurses utilized a cellphone to call the provider and complete the visit instead of using the video visit technology. 

### 3.4. Creation of Process Maps

The specific steps involved in establishing and maintaining Vet Connect are outlined in process maps developed continuously throughout the implementation process (see Figure A1 and Figure A2). We used process maps to outline Vet Connect implementation steps and to identify facilitators (Section 3.4.1) and barriers (Section 3.4.2) to implementation of Vet Connect described in more detail below. 

#### 3.4.1. Factors Facilitating Successful Program Implementation

VA leadership support and informal feedback from and regular contact between VA and non-VA partners facilitated project implementation. Frequent team communication and collaboration was necessary to adapt program components on a weekly basis. A dedicated project coordinator centralized organization of tasks and communication. 

Denver VAMC leadership support was provided by: the director of palliative care, chief of psychology, the facility telehealth coordinator, and the VA-based CNH program social work supervisor. Buy-in from these key stakeholders allowed for guidance in understanding VA policies related to obtaining appropriate technology equipment, swift onboarding of staff, and garnering provider support.

Frequent in-person, phone, and email conversations with CNH and VA staff facilitated program implementation by timely identification of gaps in access to subspecialty care and rapid program modifications to fill identified gaps. For example, an in-person meeting with a CNH transportation coordinator revealed the need for more timely specialty care referrals. In this case, we identified that newly admitted Veteran residents waited weeks to establish a primary care provider who could enter a subspecialty care referral. In fact, the need was only for the subspecialty care referral not a primary care provider because they already had a primary provider at the CNH. The Vet Connect program filled this need specifically by assigning a geriatrician as a primary point of contact who could make needed subspecialty care referrals and acts as a connection between the VA and CNHs, lending to faster referral times. This timely identification and response to identified needs facilitated rapid program implementation.

Prior relationships between providers and team nurses, who had worked at the Denver VAMC CLC and Veterans who had resided there allowed for informal communication and feedback between VA provider stakeholders and project staff. Further, weekly team meetings provide essential time for problem solving, strategizing, and incorporating feedback received from both VA and non-VA partners. 

#### 3.4.2. Barriers to Successful Program Implementation

Administrative tasks including the development of a scheduling system for clinics proved complex and the timeline for building clinics took longer than initially expected. Continuous communication with VA telehealth and Health Administrative Systems (HAS) staff was necessary to successfully build clinics for each subspecialist. Furthermore, allotting appropriate workload credit to providers proved difficult in the Denver VAMC system for similar reasons, as adding or modifying new clinics for providers, again, took time and persistent follow-up. Given that workload credit directly contributes to provider performance reporting, accuracy is essential to accurately capture providers’ workload credit and thus proved to be a key step to clarify. As one of the first VA to non-VA facility telehealth programs in the Denver VAMC, novelty of the program necessitated increased need for team problem solving and collaboration with facility partners to develop tailored processes and documentation. Building relationships with the CNHs proved challenging, due to high turnover of staff managing multiple collateral duties leading them to be overburdened [22]. One other barrier we identified included Veterans having trouble hearing providers during visits, due to hearing impairments and, in some cases, technical connection issues.

## 4. Discussion

Video visits between CNHs and VA providers are feasible based on completion of 203 visits across three subspecialties in 11 different CNHs, with technical success in the majority (83%) of visits over the 11-month implementation period of the Vet Connect project. Given that cost savings for CNHs are estimated at $160 and $150 for VA, based on substitution of a video visit for an in-person visit, a focus on sustaining the program and overcoming the barriers identified is justified. 

As the program has proved, sustainability will be a critical next step. This is especially true because such video visit referrals are becoming more common in the delivery of health care nationally [23]. Sustainability will likely depend on continuing to fill gaps in care and development of a compelling business case based on cost savings. Vet Connect originally offered geriatrics and palliative care services, but we quickly identified mental health services as an important unmet need. The demand for mental health services became evident through the number of visits and follow-up visits utilized (indicated in Figure 1). This need for mental health care services for CNH Veterans is reflective of the increasing demand for Veteran mental health care overall provided by the VA [24]. Therefore, the sustainability and expansion of programs like Vet Connect may be even more critical for efficient and improved access to care. Additionally, given that our initial cost analyses indicate that this project will have cost saving implications at both VA and non-VA facilities, engaging in the expansion of this project and similar projects could be fiscally advantageous particularly if programs like Vet Connect are adopted system wide in the VA. 

Technical metrics indicate that CVT tablets utilizing Jabber software are a reliable medium to host video visits [20]. Poor connectivity is more common in rural locations and in facilities with low bandwidth. New telehealth software, called VA Video Connect [25], has become available and may have even better functionality. While this technology has not been implemented in Vet Connect, we are training CNH staff how to use it. Other technical adjustments include the purchase of headphones with ear and mouth pieces to help veterans better participate in their appointments when they experience hearing impairment. We identified lessons learned from Vet Connect through process mapping to better understand program implementation barriers and facilitators [26]. Firstly, allocating adequate time and staffing to navigate administrative processes such as clinic creation, staff training, and care coordination is essential. Secondly, we recommend meeting with hospital support staff early to determine realistic plans and timelines. Thirdly, providing technical assistance and appropriate training to key stakeholders is critical for telehealth program uptake, as demonstrated by Vet Connect and previous community palliative care, psychiatry, and multidisciplinary telehealth programs [27,28,29]. This technical support may be best executed by hiring a dedicated project staff member to ensure successful rollout. Fourthly, we recommend having frequent team meetings for program adaptation to troubleshoot barriers to implementation and project success as rapidly as possible. Finally, as previous telehealth programs have found [28,29], and as we learned, buy-in from providers is critical, as their commitment of time and effort to use a new healthcare modality is the cornerstone of these programs.

Limitations of this project’s methodology include sample size and assumptions made in our initial cost analyses. Firstly, savings only occur if the video visit is substituted for an actual face to face visit, which may not always be the case. Secondly, Vet Connect visits often increased access to care, in that video visits increased the number of subsequent visits and referrals to the VA for CNH Veterans. It is important to note these assumptions in cost analyses. However, future research will attempt to answer whether Vet Connect saves costs longitudinally, given that increased access to care may cost more in the short-term and pay off in the long-term in lower rates of hospitalization [11]. Lastly, given the small sample size of this analysis, further work is necessary to assess the generalizability of these cost estimates. 

Future directions for this project include conducting qualitative interviews with partnered VA specialty care providers, project team nurses and staff, and non-VA facility staff to better understand facilitators and barriers of the program from their perspectives. Future qualitative publications from these interviews will also shed light on potential for program sustainability and expansion. We also plan to disseminate lessons learned about facilitators and barriers of Vet Connect implementation to ideally influence best practices nationally. While Vet Connect illustrates many VA-specific steps, implementation lessons learned can inform non-VA health care entities, particularly those serving rural areas with multiple nursing homes or outpatient clinics who have interest in implementing a similarly focused geriatric telehealth model. Referencing our process maps can inform their efforts. Given the growing number of elderly Veterans and citizens in need of long-term care and an uptake in use of telehealth nationally, programs like Vet Connect will be useful in offering two, timely, and cost-effective specialty care while saving travel time to main medical facilities.
